# Task Roadmaps: Speeding up Task Replanning

**DOI:** 10.3389/frobt.2022.816355

**Published:** 2022-04-28

**Authors:** Anders Lager, Giacomo Spampinato, Alessandro V. Papadopoulos, Thomas Nolte

**Affiliations:** ^1^ Mälardalen University, Västerås, Sweden; ^2^ ABB AB, Västerås, Sweden

**Keywords:** autonomous robots, task planning, optimization, ROS, robot task modelling

## Abstract

Modern industrial robots are increasingly deployed in dynamic environments, where unpredictable events are expected to impact the robot’s operation. Under these conditions, runtime task replanning is required to avoid failures and unnecessary stops, while keeping up productivity. Task replanning is a long-sighted complement to path replanning, which is mostly concerned with avoiding unexpected obstacles that can lead to potentially unsafe situations. This paper focuses on task replanning as a way to dynamically adjust the robot behaviour to the continuously evolving environment in which it is deployed. Analogously to probabilistic roadmaps used in path planning, we propose the concept of *Task roadmaps* as a method to replan tasks by leveraging an offline generated search space. A graph-based model of the robot application is converted to a task scheduling problem to be solved by a proposed Branch and Bound (B&B) approach and two benchmark approaches: Mixed Integer Linear Programming (MILP) and Planning Domain Definition Language (PDDL). The B&B approach is proposed to compute the task roadmap, which is then reused to replan for unforeseeable events. The optimality and efficiency of this replanning approach are demonstrated in a simulation-based experiment with a mobile manipulator in a kitting application. In this study, the proposed B&B Task Roadmap replanning approach is significantly faster than a MILP solver and a PDDL based planner.

## 1 Introduction

With the introduction of stationary and mobile robots in collaborative settings (Ajoudani et al., 2018), robots need a more sophisticated autonomous behaviour to handle an increasingly dynamic environment both safely and efficiently. Robots must be capable of dealing with such uncertainty at runtime, without impacting too much on their expected productivity. The path planning problem has been extensively discussed in the literature (Kavraki et al., 1996; Mac et al., 2016; Costa and Silva, 2019; Mannucci et al., 2019; Tajvar et al., 2020) as one important aspect to be able to guarantee a safe operation of the robot, and avoid collision with humans, robots, or other unexpected objects present in the environment. However, an efficient feasible path may not be easy to find at runtime, e.g., due to physical constraints of the environment, and the robot may need to stop waiting for the path to be cleared or make an extended detour. Whenever an unforeseeable event is perceived, e.g., the robot path is not cleared, or a task exception occurs, a task replanner can re-assign the sequence of tasks to the robot to keep its productivity high (Lager et al., 2019).

In this paper, we propose a *task planning* approach for industrial robots and service robots, called *Task Roadmaps* (TRM), that can be used for replanning the robot’s task allocation at runtime. The approach is inspired by Probabilistic Roadmaps (Kavraki et al., 1996), as it uses a similar idea to speed up the replanning of tasks at runtime. An initial plan may be generated offline while replanning is an online activity that has a direct impact on productivity, as well as the perceived reactive responsiveness of a robot.

In this work, the TRM approach is applied to a robot application modelled in the form of a Robot Task Scheduling Graph (RTSG). RTSG is an intuitive graph-based task modelling formalism for robot applications in dynamic environments that was proposed in our previous work (Lager et al., 2021). An RTSG model can be converted to a mathematical representation of the related task scheduling problem as a Mixed Integer Linear Programming (MILP) problem. The solution of the MILP problem provides the execution sequence of tasks to complete the mission with a minimized makespan. Additionally, an RTSG model can be converted to a domain and problem description in the Planning Domain Definition Language (PDDL), allowing for the scheduling problem to be solved by planners compatible with this format.

Unfortunately, the MILP formulation is an NP-hard problem (Miloradović et al., 2020; Miloradović et al., 2021), and computing a solution can be time-consuming. Compared to MILP solvers, PDDL based planners tend to be more efficient for RTSG models with more constraints but less efficient for models with fewer constraints (Lager et al., 2021).

In this paper, we propose the concept of TRM and present a Branch and Bound (B&B) algorithm to solve the very same scheduling problem described above while generating a reusable planning space (a task roadmap). Whenever replanning is needed, the B&B algorithm can leverage the planning space, which will speed up the replanning time considerably. This usage scenario of the algorithm is referred to as B&B-TRM.

In a simulation-based experimental study, we compare the replanning performance for a MILP solver, a PDDL planner, B&B, and B&B-TRM in a kitting application with a mobile manipulator. The experiments show a significant reduction of task replanning time with B&B-TRM compared to the other approaches, while providing equivalent solutions in terms of cost.

The remainder of this paper is organized as follows. Section 2 presents related works, Section 4 gives an introduction to the task modelling formalism, RTSG, and the general scheduling problem. Section 4 details the scheduling problem formulation as a MILP, Section 5 shows how RTSG can be converted to PDDL. Section 6 introduces Task Roadmaps, exemplified with a B&B scheduling algorithm for RTSG models. Section 7 presents the experimental results, while Section 8 concludes the paper.

## 2 Related Work

Some replanning approaches make a new plan from scratch when an unexpected condition occurs, e.g., see the work by Weser et al. (2010). This is a solid approach for high-quality plans but often at a high price of computational time for large problem instances. Moreover, our approach essentially makes a replanning from scratch but in addition, it leverages the search space generated to find the initial plan, thereby reducing the planning time.

Other approaches try to reuse the initially generated *plan*, modifying parts of it to adapt to unexpectedly changed or more refined conditions. The purpose can be to locally optimize the initially planned sequence, e.g., with rule-based transformational planning (Kazhoyan et al., 2020) or by rearranging subgoals at runtime using Hierarchical Planning (Hadfield-Menell et al., 2013). The purpose can also be to repair a plan, e.g., by making a rule-based rearrangement of operations (Lou et al., 2012). This way of replanning can be more simple and efficient than replanning from scratch, but the quality of a modified plan may become less optimal or invalid (McDermott, 1992). A sophisticated variant of this approach creates an adaptable and partially ordered initial plan, having an online algorithm generating a set of completely ordered plans and dispatching the one with the best chance for success given the current state (Lima et al., 2020).

The Traveling Salesperson Problem (TSP) with Precedence Constraints (PCs) with a fixed starting- and endpoint is a special case of the scheduling problem for RTSG models targeted in this work. RTSG models additionally include alternative sequences and interrupt locks. One example of a TSP-PC problem instance is TSP with pickup and delivery (Dumitrescu et al., 2009). Recently, a dynamic programming approach to solve TSP-PC dating back to 1979 was revisited (Salii, 2019). In this approach, which is akin to our proposed B&B (that uses a breadth-first and forward search approach), the algorithm starts from an empty set of nodes and uses an expansion operator to select the order-theoretic minimal of the remaining nodes in every iteration.

## 3 Task Modelling Formalism and Scheduling Problem Formulation

In this section, RTSG, the task modelling formalism used in this paper, is presented. This is followed by a description of the task scheduling problem and related assumptions.

### 3.1 Robot Task Scheduling Graph

**FIGURE 1:**
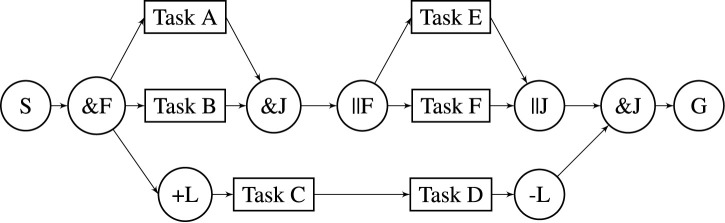
Robot task scheduling graph.

An RTSG is a directed acyclic graph, as exemplified in Figure 1. The graph is composed, e.g. by a domain expert, to specify the variability of a task sequence from a start node (S) to a goal node (G) that will achieve a higher-level goal, e.g., to fetch and deliver a selection of different objects from a warehouse. S has one outgoing edge and G has one incoming edge. Intermediate nodes in rectangular form represent tasks that may be executed in a scheduled sequence to reach the goal. Tasks have one incoming and one outgoing edge and represent robot actions at different locations in the environment, e.g. the fetching of an object. Edges and paths (of edges) represent precedence constraints. For example, if there is a directed path from task A to task B, then task A must precede task B in any schedule where both A and B are present. The remaining nodes, with a circular shape, are logical nodes that guide the variability of the task sequence. These are intuitively described in the next Sections 3.2–3.4

### 3.2 AND-Pairs

An *AND-pair* is an AND-Join node (&J) and a corresponding preceding AND-Fork node (&F). The AND-Fork node has a single incoming edge and multiple outgoing edges, while the AND-Join node has multiple incoming edges and a single outgoing edge. AND-pairs split a single branch into parallel branches at the AND-Fork node (&F) and rejoin them at the AND-Join node (&J).

The function of AND-pairs is to indicate more complex precedence constraints by being able to fork and rejoin branches. The mutual scheduling order of tasks in different parallel branches is variable since there is no directed path between them. Additionally, tasks in these branches must be scheduled before any task succeeding the AND-Join node.

### 3.3 OR-Pairs

An *OR-pair* is an OR-Fork node and a corresponding succeeding OR-Join node. The OR-Fork node has a single incoming edge and multiple outgoing edges while the OR-Join node has multiple incoming edges and a single outgoing edge. An OR-pair contains *alternative* branches of tasks, where at most one of them will be scheduled. If an OR-pair is contained by another OR-pair, it is said to be *internal*, otherwise, it is *external*. For an external OR-pair, one of its contained branches will be scheduled. For an internal OR-pair, one of its branches will be scheduled if the OR-pair is a part of a scheduled branch.

### 3.4 Lock-Pairs

A *Lock-pair* is an +L node and a corresponding succeeding -L node. These nodes have a single incoming edge and a single outgoing edge. The sub-graph between a Lock-pair must be scheduled uninterrupted by externally located tasks.

### 3.5 The Task Scheduling Problem

The problem to solve is to generate a sequence of tasks that minimises the cost to achieve the goal in a way that satisfies the constraints of the given RTSG model. Apart from the sequencing of tasks, a selection of alternative tasks is generally included in the scheduling problem. The cost to be minimized includes routing costs implicated by the task sequence selection.

It is a deterministic, single robot scheduling problem with non-concurrent tasks, where the task allocation type is a time extended assignment (Gerkey and Matarić, 2004). The state is fully observable at planning/replanning.

Targeted replanning scenarios handle unexpected states that are blocking or delaying the progress of task execution. They include the considering of obstacles that are obstructing the execution of the initially planned path/route or blocking the access of planned task locations. Additionally, they may include unexpected circumstances affecting the time to execute a task, e.g., when the robot needs to pick an object from a shelf location, and there are no objects in the box; the box will be eventually refilled, e.g., by a human but the completion of the task is affected by an unexpected duration. As a consequence of the replanning scenario, the cost for the initially planned sequence may increase and in the extreme case make it impracticable. The transition cost may become changed between many tasks and not only affect the currently running task or its successor. After rescheduling, the order of tasks to be executed may be changed or remain. Additionally, alternative tasks may become replaced.

Replanning for an unexpected adding of sub-goals, requiring a structural modification/extension of the RTSG model, is not investigated in this work. However, the removal of modelled sub-goals may be handled, e.g., by penalizing the cost for related tasks or with a selective pruning by the proposed B&B algorithm.

The computational complexity of the RTSG scheduling problem depends on the structure of the graph. As a simplistic example, the RTSG can be used to model two alternative branches of totally-ordered sequences of actions encapsulated by an OR-pair. The solution to this problem can be solved in polynomial time. On the other hand, RTSG can also be used to model a Traveling Sales Person problem (TSP). This is a problem that is known to be NP-hard (Grefenstette et al., 1985) indicating the general RTSG problem is at least NP-hard.

A planner supporting temporal PDDL may be used to address problems of harder complexity classes than NP, e.g. a temporal plan *existence* problem may be EXPSPACE-complete (Rintanen, 2007). Rintanen shows that a significant fragment of temporal PDDL planning problems can be reduced in polynomial time to classical planning with a complexity class of PSPACE. The requirements for this reduction include no overlapping of the same action and state-independent action duration. However, RTSG planning problems have state-dependent action durations. Classical domain-independent planning languages do not support state-dependent cost. However, it might be possible to reduce the problem into a classical problem by generating a manifold of fixed-cost actions (Geißer et al., 2015). The modelling approach in this work is not based on a standardized format, e.g., classical PDDL, which is too limited for the approach. Instead, it uses the native SAS+ format (Bäckström and Nebel, 1995). Despite the improvements suggested by Geißer et al., these conversions will in the worst case grow exponentially. The combination of these drawbacks for the usage of classical planners motivated the selection of a temporal PDDL planner as one of the benchmarks in this study.

## 4 Mixed Integer Linear Programming Representation

The task scheduling problem can be formulated as a Mixed Integer Linear Programming (MILP) problem where the decision variables and the constraints are derived from the RTSG model. The optimization objective is to minimize a cost function, e.g., in the form of a total completion time. The MILP problem formulation is detailed in this section.

### 4.1 Notation

A is the set of all task nodes in the RTSG, S is the start node and G is the goal node. We indicate with $A^{S}=A\cup S$ , with $A^{G}=A\cup G$ , and with $\tilde{A}=A\cup S\cup G$ .The set O⊆A , is the set of all task nodes contained by OR-pairs, thus indicating alternative tasks that may, or may not be a part of a valid task sequence.The notation j≺k where $j,k\in \tilde{A}$ indicates that task j must be scheduled before task k . The relation j≺k holds if there is a directed path from j to k in the RTSG.

### 4.2 Problem Formulation

The problem that needs to be solved is to select a set of tasks within $\tilde{A}$ and their sequence, starting from S and ending in G , subject to the constraints indicated by the RTSG so that the cost is minimized. Such a problem, can be formulated as an optimization problem, where the decision variables are $X_{j,k}\in \left\{0,1\right\}$ , $\forall j,k\in \tilde{A}$ , where

$$X_{j,k}=\left\{\begin{array}{cc} 1,\; & \text{if there is a scheduled transition from}\;j\;\;\text{to}\;\;k\\ 0,\; & \text{otherwise}. \end{array}\right.$$

Note that $X_{j,j}=0$ , $\forall j\in \tilde{A}$ since we require that there is always a transition to a different task.

The cost for selecting a transition between task j and task k is indicated with $K_{j,k}\in R_{\geq 0}$ , and it includes the transition cost $\tau_{j,k}$ , i.e., the time to move from the location of task j to the location of task k , and the time $\alpha_{k}$ that is required to complete the action of task k :

$$K_{j,k}=\tau_{j,k}+\alpha_{k}.$$ (1)

The optimization problem aims to minimize the following cost function:

$$J=\underset{j\in A^{S}}{\sum }\underset{k\in A^{G}}{\sum }X_{j,k}K_{j,k}$$ (2)

### 4.3 General Constraints

The minimization problem is subject to the following constraints.

• There is exactly one transition from, and one transition to the non-alternative nodes. However, there is no transition *from* the goal node and no transition *to* the start node:

$$\underset{k\in A^{G}}{\sum }X_{j,k}=1\;\;\forall j\in A^{S}\backslash O$$ (3)

$$\underset{k\in A^{S}}{\sum }X_{G,k}=0$$ (4)

$$\underset{j\in A^{S}}{\sum }X_{j,k}=1\;\;\forall k\in A^{G}\backslash O$$ (5)

$$\underset{j\in A^{G}}{\sum }X_{j,S}=0$$ (6)

• No cyclic sub-routes: Let V⊆A be any non-empty subset of tasks.

$$\underset{j\in V}{\sum }\underset{k\in V}{\sum }X_{j,k}\leq |V|-1\;\;\forall V\subseteq A,V\neq Ø$$ (7)

• Precedence constraints: Let $D\subseteq \tilde{A}$ be any ordered subset with multiple elements where the last element precedes the first element. $D_{i}$ is the i -th element of D .

$$\overset{|D|-1}{\underset{j=1}{\sum }}X_{D_{j},D_{j+1}}\leq |D|-2\;\forall D\subseteq \tilde{A},\;|D|\geq 2,\;D_{\left|D\right|}≺D_{1}.$$ (8)

### 4.4 Lock-Pair Definitions and Constraints

A Lock-pair contains a set of tasks, L⊆A . Tasks in L must be scheduled as a group in a sub-sequence that is uninterrupted by other tasks. Two subsets of L are defined: $L^{F}=\left\{a\in L\;|b⊀a\;\forall b\in L\right\}$ specifies the *first tasks* of L while $L^{L}=\left\{a\in L\;|a⊀b\;\forall b\in L\right\}$ specifies the *last tasks* of L.

The constraints associated with Lock-pairs restrict transitions from/to tasks contained by the pair. There can at most be one transition from external tasks to the first tasks, and at most one transition from the last tasks to external tasks:

$$X_{j,k}=0\;\;\forall L,\forall j\in L\backslash L^{L},\forall k\in A^{G}\backslash L$$ (9)

$$X_{j,k}=0\;\;\forall L,\forall j\in A^{S}\backslash L,\forall k\in L\backslash L^{F}$$ (10)

$$\underset{j\in L^{L}}{\sum }\underset{k\in A^{G}\backslash L}{\sum }X_{j,k}\leq 1\;\forall L$$ (11)

$$\underset{j\in A^{S}\backslash L}{\sum }\underset{k\in L^{F}}{\sum }X_{j,k}\leq 1\;\forall L$$ (12)

### 4.5 OR-Pair Definitions and Constraints

The OR-pair constraints presented in this section can handle OR-Pairs inside OR-Pairs etc., in a recursive manner. To express the OR-pair constraints we first need to prepare some supporting definitions and operators:

An OR-pair contains a set of *OP nodes*, where an *OP node* is either a task node or an internal OR-pair. In the same way, internal OR-pairs contain OP nodes etc.

Formally, $O_{1},\ldots ,O_{v}$ are OP node sets contained by OR-pair 1,…,v ; $O_{p1},\ldots ,O_{pm}$ , are OP node sets in branches 1,…,m of OR-pair p ; $O_{pq}^{T}=\left\{a\in O_{pq}\;|a\text{ is a task}\right\}$ contains task nodes and $O_{pq}^{OP}=\left\{a\in O_{pq}\;|a\text{ is an internal OR}-\text{Pair}\right\}$ contains internal OR-pairs so that $O_{pk}=O_{pq}^{T}\cup O_{pq}^{OP}$ .

One *primary* OP-node, $P_{pq}\in O_{pq}$ is arbitrarily selected for each OR-pair branch. We define the following operators:

• F is a recursive operator that returns a set of tasks for a given OR-pair branch. The set represents alternative tasks in the branch that shall not be combined:

$$F\left(O_{pq}\right)=\left\{\begin{array}{cc} \left\{a\right\}\; & \text{if }P_{pq}\text{ is task }a\text{.}\\ F\left(O_{r1}\right)\cup \ldots \cup F\left(O_{rm}\right)\; & \text{if }P_{pq}\text{ is OR}-\text{pair }O_{r} \end{array}\right.$$ (13)

• H returns a set of tasks for a given OR-pair. It represents alternative tasks in the OR-pair that shall not be combined:

$$H\left(O_{p}\right)=F\left(O_{p1}\right)\cup F\left(O_{p2}\right)\cup \ldots \cup F\left(O_{pm}\right)$$ (14)

• R returns a *set of sets* of tasks for a given OR-pair branch. It represents other sets (than F ) with alternative tasks in the branch that shall not be combined:

$$R\left(O_{pq}\right)=O_{pq}^{T}\backslash P_{pq}\cup {⋃}_{i\in O_{pq}^{OP}\backslash P_{pq}}\left\{H\left(i\right)\right\}$$ (15)

With these definitions, the OR-pair constraints can be summarized:

• Transitions from and to tasks contained by OR-pairs: There is at most one transition from/to such a task and the number of incoming transitions is the same as the number of outgoing transitions:

$$\underset{k\in A^{G}}{\sum }X_{j,k}\leq 1\;\;\forall j\in O$$ (16)

$$\underset{j\in A^{S}}{\sum }X_{j,k}\leq 1\;\;\forall k\in O$$ (17)

$$\underset{k\in A^{G}}{\sum }X_{j,k}=\underset{k\in A^{S}}{\sum }X_{k,j}\;\;\forall j\in O$$ (18)

• Transition from and to tasks in alternative OR-pair branches: For an external OR-pair, exactly one branch will be scheduled. For any OR-pair: If the primary OP node in one branch is scheduled, the remaining OP-nodes in the same branch will also be scheduled.

$$\underset{j\in A^{G}}{\sum }\overset{m}{\underset{q=1}{\sum }}\underset{s\in F\left(O_{pq}\right)}{\sum }X_{s,j}=1\;\;\forall \text{ external }O_{p}$$ (19)

$$\underset{j\in A^{S}}{\sum }\overset{m}{\underset{q=1}{\sum }}\underset{s\in F\left(O_{pq}\right)}{\sum }X_{j,s}=1\;\;\forall \text{ external }O_{p}$$ (20)

$$\underset{j\in A^{G}}{\sum }\underset{k\in R^{\prime }}{\sum }X_{k,j}=\underset{j\in A^{G}}{\sum }\underset{s\in F\left(O_{pq}\right)}{\sum }X_{s,j}\;\forall O_{pq},\forall R^{\prime }\in R\left(O_{pq}\right)$$ (21)

$$\underset{j\in A^{S}}{\sum }\underset{k\in R^{\prime }}{\sum }X_{j,k}=\underset{j\in A^{S}}{\sum }\underset{s\in F\left(O_{pq}\right)}{\sum }X_{j,s}\;\forall O_{pq},\forall R^{\prime }\in R\left(O_{pq}\right)$$ (22)

### 4.6 Replanning Constraints

In dynamic environments, some unforeseeable events may make the initially computed plan not possible to execute. To complete the robot mission, a replanning can be initiated. Such a replanning can, besides changing the order of tasks, exploit other OR-pair branches of the RTSG to successfully complete the mission in an alternative way. On the other hand, if one or more tasks in an OR-pair branch are already completed, the remaining tasks in this branch will also become scheduled in the new plan. For replanning, one needs to introduce additional constraints to account for completed tasks, and for capturing the changed situation that hinders the completion of the initial plan. The set $C=\left\{C_{1},\ldots ,C_{l}\right\}$ contains the sequence of already completed tasks. The initial transitions for these tasks become additional replanning constraints:

$$X_{S,C_{1}}=1$$ (23)

$$X_{C_{i},C_{i+1}}=1\;\forall i=1,\ldots ,l-1$$ (24)

Further, the costs for possible transitions between tasks, $K_{j,k}\;j\in A^{S}\backslash C,k\in A^{G}\backslash C$ are updated to describe the current situation. For example, the costs are affected by unexpected obstacles and the new location of S , i.e., the current location of the robot. Thereafter, the cost of transitions involving completed tasks are initialized:

$$\begin{array}{ll} K_{S,C_{1}} & =0\\ K_{C_{i},C_{i+1}} & =0\;\forall i=1,\ldots ,l-1\\ K_{C_{l},j} & =K_{S,j}\;\forall j\in A^{G}\backslash C \end{array}$$

## 5 Planning Domain Definition Language Representation

PDDL is a general domain-independent modelling formalism for setting up planning problems, originating from classical planning (Fikes and Nilsson, 1971). It is used to define planning problems in many areas, also outside the field of robotics. Planning problems that are represented in a PDDL format can be solved by many different planner algorithms, e.g., Temporal Fast Downward (TFD) (Eyerich et al., 2009) and POPF2 (Coles et al., 2011). Since an RTSG model can be converted to a PDDL planning problem (Lager et al., 2021), many different planner algorithms are available. One of these, TFD, is used in this work as a benchmark. The reason for selecting TFD was it is ability to find high quality solutions in comparison with other planners/solvers in our previous work (Lager et al., 2021). For compatibility, planners need to support some parts of PDDL2.1 (Fox and Long, 2003) that extends PDDL with syntax for temporal planning.

### 5.1 Planning Domain Definition Language Introduction

A PDDL problem specification is divided into a domain description and a problem description. In the domain description, definitions are made that can be reused for similar planning problems. The most basic definitions include:

• *Types* are used to instantiate different types of objects, e.g., robots, locations, tools, paths, or boxes. The types can be organized with polymorphism, e.g., a tool may be a gripper or a camera.

• *Predicates* are used to instantiate different types of facts, describing relations between objects, e.g., “robot1 is at location1″, “location1 and location2 is connected with path1”.

• *Actions* are operators that can be applied if a set of preconditions, specified as predicates, hold for a set of object parameters. The application of an action causes a set of effects, also specified as predicates, that will add or remove facts. Action example: Move robot1 from location1 to location2. Preconditions are: 1) “robot at location1″ and 2) “location1 and location2 is connected with path”. Effects are: 1) “robot is at location2″ and *not* “robot is at location1”.

A basic PDDL problem description includes:

• Existing objects of different types.

• Facts describing initial relations between objects, i.e., the initial state.

• Facts describing desired or undesired relations between objects, i.e., the goal state.

The task of a *planner algorithm* is to process the domain and problem description and find a sequence of applicable actions operating on the existing objects, that will change the initial state to a state where the goal state is fulfilled. This plan generation process is not investigated here. Instead, details are presented on how to convert the RTSG task modelling formalism into PDDL, thereby enabling plan generation with already existing planner algorithms.

### 5.2 Conversion From Robot Task Scheduling Graph to Planning Domain Definition Language

In general, when converting an RTSG model to a PDDL specification, the RTSG nodes become objects and the edges become facts. Two types of PDDL actions are defined:

• Running a task. This is a durative action where the duration is the cost to perform the task.

• Firing a transition of a logical node. The purpose of this action type is to enable the execution of tasks under the constraints imposed by the logical nodes, e.g., precedence constraints and alternative OR-pair branches. They do not correspond to real actions and their duration is zero in order not to affect the cost.

#### 5.2.1 A Planning Domain Definition Language Domain for Robot Task Scheduling Graph

Listing 1 presents a PDDL domain for a general RTSG. The domain is almost independent of the RTSG model that shall be converted.

Listing 1: PDDL domain

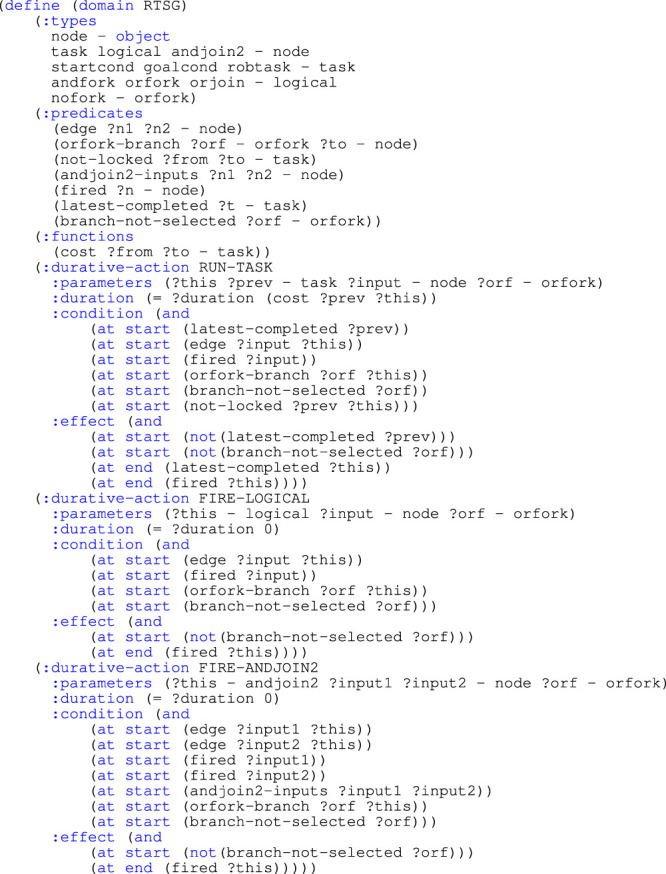

All object types are derived from the *node* type, and are used to represent nodes in an RTSG model. There are two types of *node*: *task* and *logical*. *task* represents actions or states that affects the cost of a plan: *startcond*, *goalcond* and *robtask*. *logical* represents different types of logical nodes: *andfork*, *orfork* and *orjoin*. Different *andjoin* types are defined for each number of incoming edges that needs to be represented in the RTSG model. For example, *andjoin2* represents an AND-Join with two incoming edges. A *nofork* object is a dummy object that helps in the modelling of alternative branches. Lock-pair nodes are not represented by object types; instead their constraints are modelled with *not-locked* facts.

Among the static predicates, that cannot be affected by any action, *edge* represents an edge between two nodes. *orfork-branch* specifies the outgoing connection from an OR-Fork to another node. *not-locked* specifies if a transition between two tasks is not locked. The other predicates are dynamic and can be created or removed by actions: *fired* specifies if a node (*task* or *logical*) is completed. *latest-completed* specifies if a task is the latest completed task. *branch-not-selected* specifies if an alternative branch for an OR-Fork not has been selected.

There are two types of actions: to run a task and to fire a transition for a logical node. There is one action that runs all tasks, i.e. RUN-TASK. There are a limited number of actions for logical nodes, i.e. FIRE-LOGICAL to run transitions for all *non* AND-Join nodes, FIRE-ANDJOIN2 for transitions of AND-Joins with two incoming edges, FIRE-ANDJOIN3 for 3 incoming edges etc. Additional AND-Join actions must only be defined if they exist in the RTSG model.

Running a task has a duration and the duration is represented by a *cost* function that specifies the cost of performing a task (*to*) after completing another task (*from*). The preconditions require that the node connected to the incoming edge has been fired. If this connected node is an OR-Fork, it is required that a branch not yet has been selected. It is also required that a transition from the latest completed task not is locked. The effects update the dynamic predicates: The task becomes both *fired* and the *latest-completed*. The *branch-not-selected* is removed for the task’s *orfork*.

Fire a transition for a logical node has a zero duration, i.e., free of cost. The preconditions require that the node connected to the incoming edge has been fired. If this connected node is an OR-Fork, it is required that a branch not yet has been selected. The effects update the dynamic predicates: The logical node becomes *fired* and the *branch-not-selected* is removed for the logical node’s *orfork*. For an AND-Join action, the preconditions additionally require *every* node connected to the incoming edges to be fired.

#### 5.2.2 A Planning Domain Definition Language Problem for an Robot Task Scheduling Graph Model

Listing 2 exemplifies a PDDL problem description at initial planning, that is converted from the RTSG model in Figure 1. Some of the data in the conversion are left out, indicated with ’ $...^{\prime }$ , to get a more compact overview.

Listing 2. PDDL problem

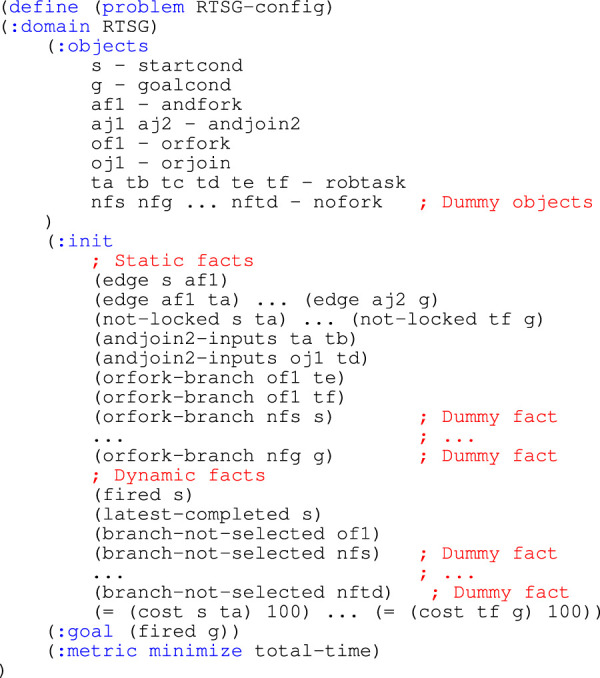

The objects and the static facts listed in the PDDL problem are dependent on the structure of the RTSG model.

Objects are defined for all nodes in the RTSG graph except for Lock-pair nodes. Additionally, *nofork* objects are created for all nodes that do not have an incoming edge from an OR-Fork node. *noforks* are dummy objects assisting in the selection of alternative OR-Fork branches.

Static facts are created to represent the structural elements of the RTSG model. The edges are represented with *edge* facts that specify connected nodes. However, edges to/from Lock-Pair nodes are bypassed. *not-locked* facts are created for each valid transition between two tasks, with respect to precedence constraints as well as Lock-pair constraints. For each AND-Join node, an *andjoinX-inputs* fact is created that specifies all nodes connected to its X incoming edges. For each node connected to the outgoing edge of an OR-Fork node, an *orfork-branch* fact is created indicating this *orfork*. For all other nodes, an *orfork-branch* fact is created indicating their *nofork* object.

Dynamic facts are created to represent all possible intermediate states of a task execution sequence. A *fired* fact is added for all completed nodes. For the initial state, as illustrated in Listing 2, there is only a single *fired* fact for the start node. At replanning, a *fired* fact is additionally added for all completed tasks and for the logical nodes that precede completed tasks. A single *latest-completed* fact specifies the latest completed task. At initial planning, the start node is always the *latest-completed*, while this may have changed at replanning. For the initial plan, a *branch-not-selected* fact is created for each *orfork* and nofork. At replanning, these facts are removed if the *orfork* has a *fired* fact or if the node that is connected to the *nofork* has a *fired* fact.

Finally, the numerical cost value for all *not-locked* transitions between tasks is specified. In a replanning scenario, these values may become changed by the current state of the modelled application.

The required goal state is a *fired* fact for the goal object. The objective of the plan, the *metric*, is to minimize the makespan.

## 6 Task Roadmaps

We developed a Branch and Bound (B&B) algorithm, to compute solutions of scheduling problems modelled with RTSG as detailed in Section 4. The algorithm makes a breadth-first expansion of a search tree. It is a forward search that is guided from the start node of the RTSG model. When replanning is needed, the algorithm is designed to make a new plan while considering the current conditions, e.g., the location of the robot and the sequence of already completed tasks. Optionally, the search space that was constructed while generating the initial sequence can be reused. This option avoids the need of expanding a new search space from scratch, making the replanning significantly faster with preserved quality of generated plans.

In analogy with Probabilistic Roadmaps (Kavraki et al., 1996), the search space is referred to as the Task Roadmap (TRM) that is created in a *learning phase* and used for runtime replanning in a *query phase*. In Table 1, we compare the basic characteristics between Probabilistic Roadmaps (PRM) and TRM.

**TABLE 1:** Similarities of the basic characteristics for Probabilistic Roadmaps and Task Roadmaps, respectively.

Probabilistic roadmaps	Task roadmaps
Represents a robot configuration space with a graph	Represents a robot task sequence space with a search tree
Used for *path* planning with obstacle avoidance	Used for *task* planning with scheduling constraints
The graph is built using probabilistic sampling of the robot configuration space	The search tree is built with a deterministic B&B algorithm where the scheduling constraints are specified with an RTSG model
Nodes represent collision-free configurations in the configuration space	Nodes represent valid task sequences with respect to the scheduling constraints
Edges represent collision-free paths between robot configurations. Path costs are typically fixed	Edges represent valid transitions that extend task sequences with applicable tasks. Transition costs are updated for a new replanning scenario
Offline learning phase to build the graph	Offline learning phase to build the search tree
Online query phase where the graph is used, e.g., at replanning, to identify a collision-free and potentially efficient path from a current state to a goal state	Online query phase where the search tree is used, e.g. at replanning to identify a valid and potentially optimal task sequence from a current state to a goal state

Additionally, a saved Task Roadmap may be leveraged to speed up *initial* planning for an RTSG model if it has the same graph structure as the model used to generate the Task Roadmap.

### 6.1 Learning Phase

**FIGURE 2:**
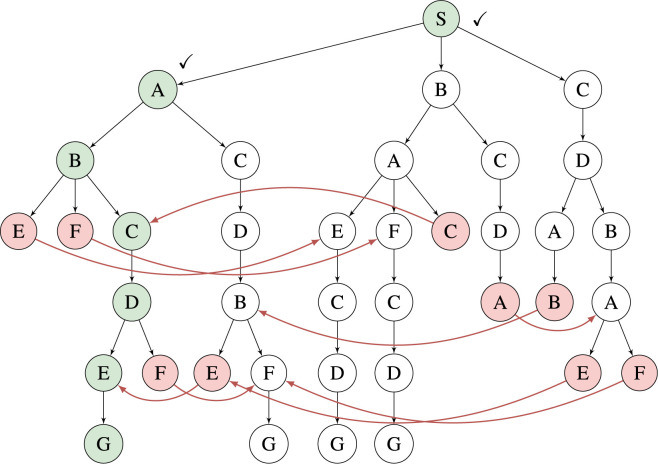
Visualization of a B&B search tree for the RTSG model in Figure 1. This expanded search tree makes up the Task Roadmap. The initial task sequence is marked in green and completed nodes have a checkmark. Pruned nodes are red and have red pruning edges.

In the learning phase, a search tree is expanded by the B&B algorithm acting on a Robot Task Scheduling Graph (RTSG) to find an initial task sequence.In the search tree, see Figure 2, the initial start condition is represented by the root node S , and other nodes represent sub-sequences. The number of tasks in a sub-sequence corresponds to the distance between the node and the root node. The best sequence is the sequence reaching the leaf G with the lowest cost (indicated in green in the figure). An important aspect of the scheduling algorithm is the pruning of equivalent nodes. Equivalent nodes have the same distance to the root node and the same combination of tasks (but in different orders) where the last task is the same, leading to a similar state. In the example in Figure 2, the two nodes representing the sequences S - A - B - C and S - B - A - C are equivalent. The difference between equivalent nodes is mainly the cost of the respective sequence leading to this state. The possible propagation of task sequences from equivalent nodes is identical. This conclusion is not formally proved here but verified experimentally in the scenarios presented in Section 7 where B&B always finds valid and potentially optimal solutions with the same objective value as a MILP solver and a PDDL planner, at initial planning as well as at replanning in the query phase. The identical task sequence propagation from equivalent nodes is leveraged in the query phase. For this purpose, the algorithm always adds a *pruning edge* from a pruned node to the equivalent node with a better cost that caused the pruning. In the following description, equivalent nodes that are interconnected with pruning edges are referred to as *peers*.

### 6.2 Query Phase

At a point of replanning, the world is in a new state where the robot has completed 0 to |A| tasks of the initial sequence. It is expected that the transition costs and the action costs of the remaining tasks (Eq. 1) may have changed, e.g., since the previously planned path to a task may have become temporarily blocked. Especially, the transition cost from the latest completed task to other tasks should be guided by the current state of the robot, e.g. its location. The query phase tries to identify an efficient sequence of tasks that will bring the robot from the current world state to the goal.

The query phase is divided into 2 steps:

1) The first step is to identify the *current node* of the search tree. This is the node that represents the sequence of already completed tasks. If no tasks have been completed, the root node becomes the current node.
2) The second step is to find the most efficient task sequence between the current node and a goal-reaching node in the search tree. In this step, pruning edges are leveraged to explore nodes without children.

When replanning is made while reusing an existing search tree, we will refer to this method as B&B-TRM. And when replanning is made from scratch with a single and non-expanded root node, we will refer to this method as B&B. The memory required for replanning with B&B-TRM is always the same amount as required by B&B to find the initial task sequence in the learning phase and create the Task Roadmap. Replanning with B&B will in worst case require the same amount of memory as B&B-TRM, i.e., if no tasks are completed. If some tasks are already completed the memory need becomes reduced due to the smaller problem size. Both methods are realized with the same algorithm. This algorithm takes as input a list of already completed tasks. It also needs cost estimates for actions and transitions with respect to the current state. The search starts from the root node, which may be an initial single node or the top node of an existing search tree. It is a breadth-first search where any existing children are reused instead of being created while expanding the search. It is pruning all nodes in the first generation except the one that matches the first completed task. Then all nodes in the second generation are pruned except the one that matches the second completed task etc. This goes on until the current node is reached. From this node and onwards, only equivalent nodes are pruned. If an expansion is required from a node without children, the algorithm will look for a peer of the node. If no peer is found (e.g., since the algorithm is run without a TRM), all applicable children of the node are identified by a search of the RTSG and created. If instead a peer *is* found, the peer’s children are adopted by this node. If the peer lacks children, the peer’s peer’s children are adopted etc. These adopted children are top nodes of sub-trees that are disconnected from the peer and reconnected to the new parent node. The cost of all reused children adopted or previously existing, need to be updated to consider the new cost of the parent. The search continues until the goal is reached in all active (non-pruned) search nodes. Finally, the goal-reaching node with the lowest cost is identified.

Due to the exchange of subtrees between peers, each replanning will potentially modify the structure of the search tree. However, no information is lost that is required for later replanning. The significant reduction of planning time from reusing nodes comes from 1) removing the process of searching the RTSG to find possible search tree propagations and 2) removing the process of creating nodes from scratch.

### 6.3 B&B and B&B-TRM

A pseudo-code for B&B and B&B-TRM is given in Algorithm 1.

Algorithm 1: B&B and B&B-TRM.

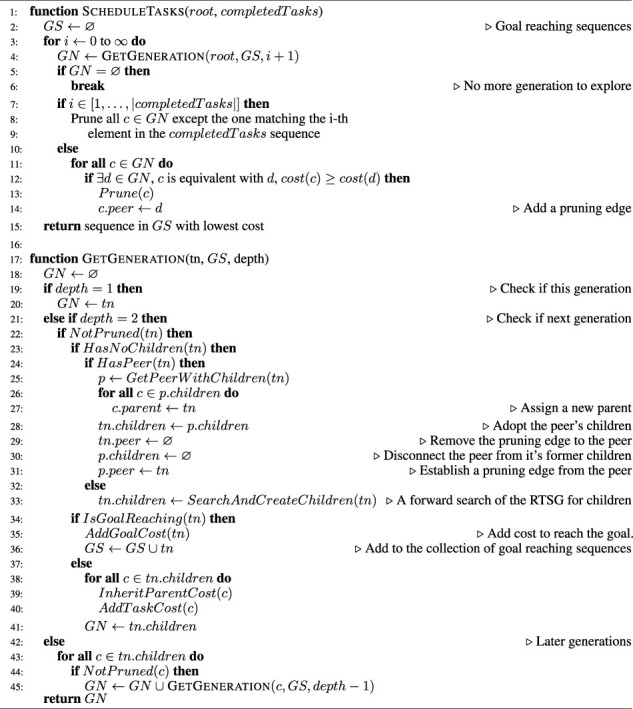

The algorithm starts at the *ScheduleTasks* function. It takes as input arguments a *root* node and a list of already completed tasks. The root node keeps the starting conditions for planning, e.g., the current location of the robot. At initial planning, the root node does not have any children. After completing the initial planning, it has been expanded to a search tree. At replanning, the expanded root node can be used as an input argument to *ScheduleTasks*, thereby speeding up the planning time. This usage scenario is referred to as the B&B-TRM algorithm. If instead a non-expanded root node is used, the search tree will be generated from scratch, which is referred to as the B&B algorithm.

*ScheduleTasks* runs a loop where a new generation of nodes is fully explored in every cycle, starting from the root node. From each generation, any goal-reaching nodes (sequences) are collected. The loop continues until no more generations can be explored. Finally, *ScheduleTasks* returns the goal-reaching sequence with the lowest cost. For each explored generation, pruning is made among equivalent nodes (in the algorithm referred to as peers ) and pruning edges (object references to peers) are recorded for all pruned nodes to keep track of reusable sub-trees. If there are completed tasks, all children of the first generations, except the ones matching the completed sequence, are pruned.

To explore a new generation, the recursive *GetGeneration* function is used. The first argument, tn , is a reference to a tree node. The function returns a list of nodes representing a generation with respect to the tree node. The generation is specified with a relative depth argument, where the value of 1 specifies the current generation, i.e., the tree node itself. A value of 2 indicates the children of the tree node while 3 indicates the grandchildren etc. If the grandchildren or later generations are specified, intermediate generations have already been explored due to the breadth-first search approach, and the existing children are used to explore the specified generation recursively. If instead the children generation is specified, the way of exploration will depend on the usage scenario. For the B&B scenario, where a reusable roadmap is missing, the children of the tree node are identified with the method *SearchAndCreateChildren*. This is a method that searches the RTSG model recursively, where any possible child nodes are created, configured, cost estimated and attached to the search tree. For B&B-TRM, existing children can be reused and if a tree node does not have children, these can be localized from its peer. If the peer has no children, the peer’s peer is checked etc until children are found. Thereafter, the children are reconnected from the peer to the tree node. For both usage scenarios, the costs of the children are estimated as the cost of the parent plus the cost to perform the last task of the child with respect to the last task of the parent. Apart from returning a list of a generation, *GetGeneration* also updates the list of goal-reaching sequences, i.e. the GS argument, with any goal-reaching sequence.

## 7 Results

### 7.1 Use Case

**FIGURE 3:**
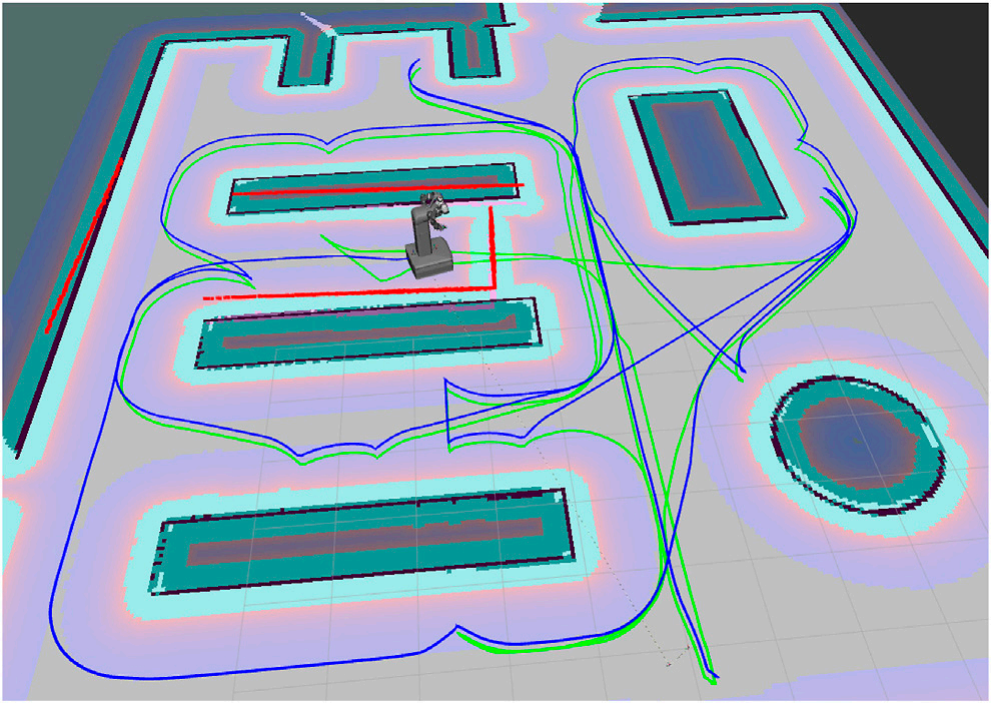
Warehouse layout. The light green path, starting and ending at the bottom right, is the initial route for Scenario **(B)**. The robot has stopped in front of an obstacle that is blocking the initially planned movement. The red lines along the shelves, the wall and the obstacle indicate the objects perceived by the robot’s laser scanner. A replanned route in blue, starting at the robot’s location, has changed the order of the remaining tasks.

The use case is a kitting application in a warehouse. A mobile manipulator shall deliver kit boxes filled with several specified objects to a delivery station. Objects and empty kit boxes are located on 5 different shelves in a simple warehouse as illustrated in Figure 3.

**FIGURE 4:**
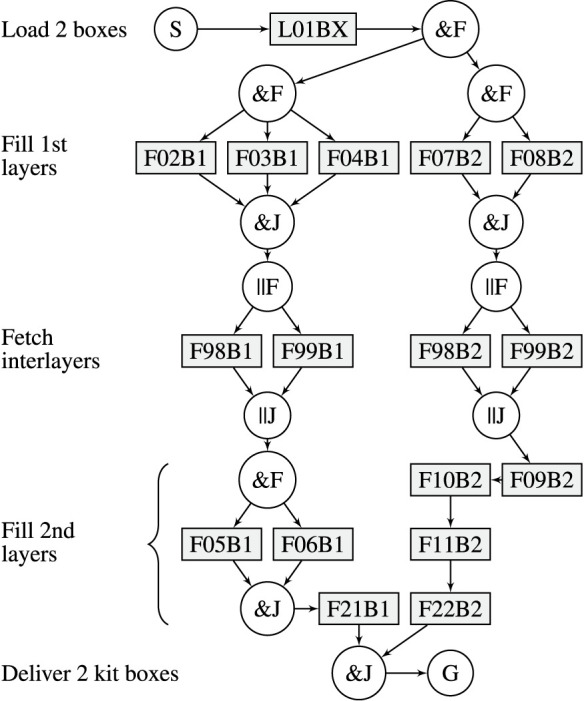
RTSG representation of Scenario A.

**FIGURE 5:**
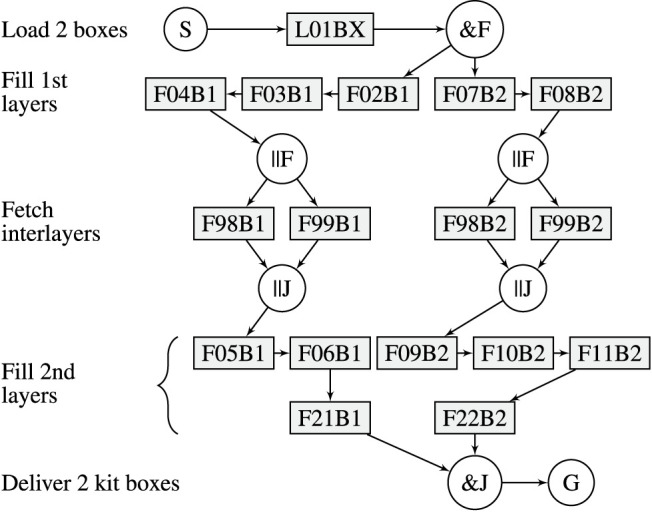
RTSG representation of Scenario B.

**FIGURE 6:**
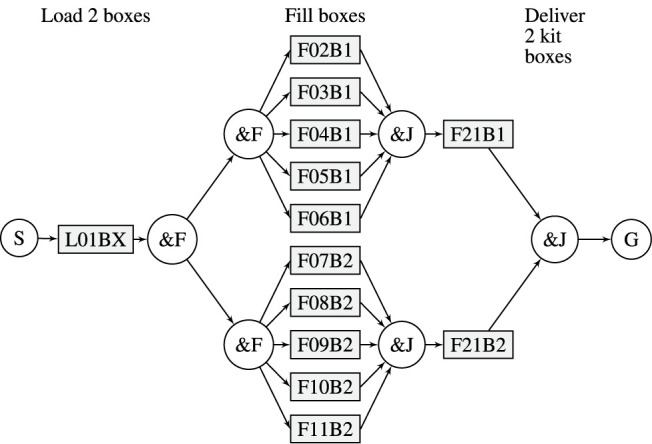
RTSG representation of Scenario C.

The robot can carry 2 kit boxes and fill them in parallel. The process to deliver the kits is divided into 5 phases: *Fetch empty kit boxes*, *Fetch layer 1*, *Fetch interlayer*, *Fetch layer 2*, and *Deliver kits*. There are two types of tasks: The first is to load empty kit boxes, and the second is to fetch an object to a carried kit box. Three different scenarios (A, B and C) are modelled with RTSG, see Figures 4–6. Task names, e.g., F98B1 , indicate type of task ( F etch), location (98) and kit box (1). In all scenarios, the first task, L01BX , is to load two kit boxes from a shelf location (01). The remaining tasks require fetching one object at a shelf location into one of the two carried kit boxes. The first AND-pair splits the graph into two branches, each one modelling the filling of one kit box. The goal node represents the movement of the robot to the goal location where the filled kit boxes shall be delivered. Scenario A is a realistic specification for how kit boxes can be filled in an industrial context. For layer one, the kit objects can be fetched in any order. For layer 2, the right-hand side kit box is modelled to be filled in strict order ( F09B2 - F10B2 - F11B2 ) while the left-hand side kit box provides some variability. A strict order can be desired, e.g., to achieve a predefined overlap of objects in the kit box. There are two alternative locations, 98 and 99, where an interlayer can be fetched. In Scenario B, tasks from Scenario A are rearranged in the graph, applying a strict order for each layer, thereby introducing more precedence constraints that reduce the variability of the task sequence. In Scenario C, the tasks are rearranged to minimize the number of precedence constraints, thereby maximizing the variability. Scenario C is closer to a TSP problem with no precedence constraints among the objects that shall be placed in the boxes.

When converting the three scenarios to MILP problem formulations, the number of variables and constraints are indicated in Table 2. Additionally, some lazy constraints are added dynamically when required for Eqs. 7 and 8 while running the MILP solver. The theoretical number of constraints for these equations can be very high and defining them all may not be viable.

**TABLE 2:** Variables and constraints for the MILP problem formulations.

Scenario	#Variables	#Constraints
A	342	46
B	342	46
C	210	30

### 7.2 Experimental Setup

The simple warehouse world is modelled with the Gazebo simulator (Koenig and Howard, 2004). This includes the shelves, the mobile robot and an obstacle that may interfere with the robot path. ROS Navigation Stack (Guimarães et al., 2016) is used to navigate the robot between different locations. The navigation is guided by a 2D map of the warehouse that initially does not include the obstacle. While navigating, the robot simultaneously maps changes in the simulated world with respect to the map.

The benchmarked planners include the proposed B&B and B&B-TRM algorithms, a MILP solver (Gurobi Optimization, 2021) and Temporal Fast Downward (TFD) (Eyerich et al., 2009) which is a PDDL based temporal planner. An initial task sequence is generated from the RTSG model with B&B, MILP and TFD. Additionally, B&B generates a task roadmap that is reused by B&B-TRM in all replanning experiments. The transition costs between tasks are represented by the collision-free path lengths generated with the Dijkstra algorithm from the initial map. The initial task sequence corresponds to a route in the warehouse that starts and ends at the same location. In the simulation-based replanning experiments, the robot navigates the initially computed route, which turned out to be the very same route for all planners, while simultaneously mapping the environment with a simulated laser scanner. When the first task is reached, the next task is dispatched etc. During the progress of the plan execution, the planned motion to the next task becomes blocked by the obstacle at randomized locations along the path. The part of the obstacle that is visible from the robot while approaching it and stopping a short distance nearby (1–1.5 m), becomes included in the map. A replanning is initiated from a randomized location of the robot in front of the obstacle on the planned path. For this purpose, the initial planning problem is updated to become a replanning problem, considering already completed tasks, the robot’s (randomized) current location and updated transition costs caused by the updated map. Depending on the location of the obstacle and its effect on collision-free path lengths between tasks, the replanned routes may change or keep the sequence of tasks. Replanning may fail if the location of the obstacle blocks a planned task when there are no alternative tasks. If a collision-free path can not be found between two tasks, the transition cost is penalized to a very high value that will help to detect a failed plan. All replanning scenarios were run with the benchmarked planners, including B&B-TRM and B&B (without the TRM). The number of already completed tasks at replanning, l , was in the range l∈{0,1,…,|A|} . 50 replannings were made for each l , with randomized robot and obstacle locations along the path between the location of the latest completed task and the next task. All experiments were run on the same computer with an Intel i5-4,570, quadcore, 64-bit processor having 7.6 GB RAM running on Ubuntu 18.04.5. For the MILP solver, the no-cyclic-subroutes constraints and the precedence constraints were implemented as lazy constraints in a customized Python callback routine. The B&B and the B&B-TRM algorithms were implemented in Python.

### 7.3 Experimental results

**FIGURE 7:**
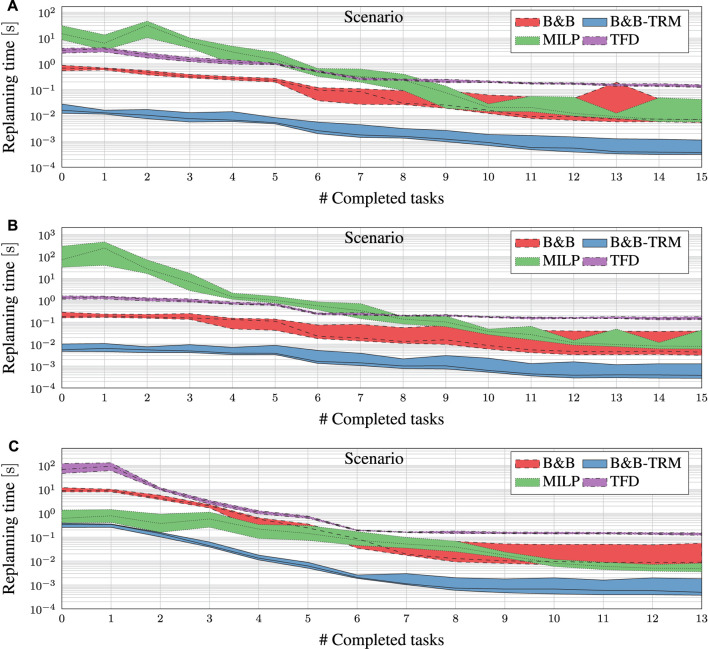
Replanning time for B&B (dashed area), B&B-TRM (solid area) and MILP (dotted area) at different task completion levels. The central line in each area indicates the median. Note that the vertical axis is logarithmic.

Figure 7 shows the experimental results for the four planner algorithms, i.e., B&B, B&B-TRM, TFD and the MILP solver. The graphs show the minimum, the median, and the maximum replanning time for the four algorithms, as a function of the number of completed tasks l ; if l is 0, no task has been completed, and the replanning is performed with all the tasks in the task set, and therefore the replanning time is expected to be higher.

For each experiment in all scenarios, B&B-TRM and B&B reach the very same objective value as the solution computed by the MILP solver, suggesting that the proposed algorithms may be optimal. However, further investigation on the optimality of the proposed algorithms is needed. TFD also reaches the very same objective values in all experiments. The replanning time for B&B-TRM is less than 1 s (and often a fraction of that) for all scenarios. As expected from B&B-TRM essentially being a reduced version of B&B, it outperforms B&B in every scenario by an average factor of 26. The factor tends to increase slightly for scaled-up problem instances with fewer completed tasks.

B&B-TRM outperforms the MILP solver in Scenario A by a factor greater than 152 for the largest problem instances l∈{0,...,8} . The difference is slightly less for the smaller problem instances but still significant (>18) . For Scenario B, B&B-TRM is more than 137 times faster for l∈{0,...,8} . For Scenario C and l∈{0,1,2} , B&B-TRM is only 1.8, 2.3 and 2.6 times faster than the MILP solver, but for l>2 , the proposed method exhibits significantly better performance. The decreasing difference to the MILP solver for the larger problem instances in Scenario C is correlated with a fast-growing search tree and increased memory consumption. For Scenario A and B, the growth of the search tree at each breadth-first expansion step is limited by the more numerous precedence constraints, thereby reducing the memory consumption considerably. Scenario C highlights the main limitation of the proposed approach, i.e., for loosely constrained problems (Figure 6), the number of alternatives grow significantly in breadth-first tree-based search approaches. On the other hand, the specification of robot applications is typically closer to Scenario A (Figure 4), with a more structured sequence of tasks, hence with additional constraints. In such cases, the proposed approach can provide a significant speedup.

B&B-TRM outperforms TFD by a factor greater than 149 for all l∈{0,...,|A|} in Scenario A. This factor is 151 for Scenario B and 68 for Scenario C, respectively. A part of this performance difference may depend on the fact that the proposed algorithm is designed for the RTSG modelling formalism used to model these scenarios, while TFD is designed for the PDDL modelling formalism.

Finally, it is worth noticing that both the B&B-TRM approach and TFD provide a more predictable performance than MILP, highlighted by the limited variability (standard deviation) of the replanning time.

### 7.4 Scalability Investigation

**FIGURE 8:**
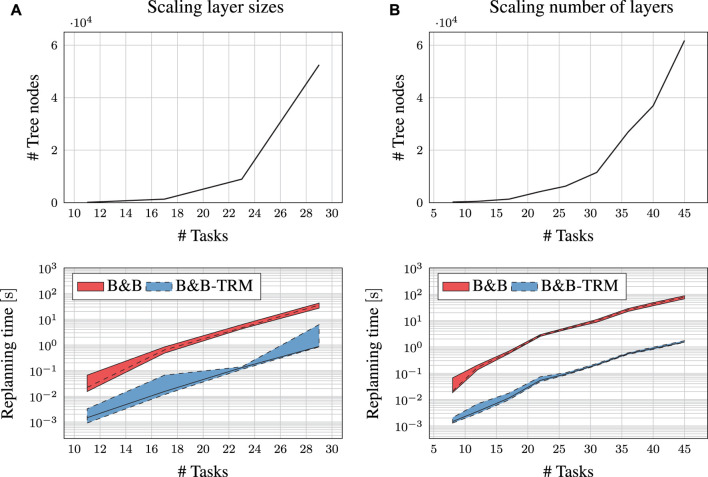
Scalability experiments for Scenario A. ‘#Tasks’ indicates the number of tasks in the RTSG. ‘#Tree nodes’ indicates the number of nodes in the Task Roadmap search tree.

As discussed in Section 3.5, the complexity of the scheduling problem depends on the structure of the graph. It is assumed that the scalability of the presented algorithm also depends on this structure, and how the graph is scaled. In order to investigate how the algorithm may scale for growing problem sizes, an experiment was setup targeting the graph in scenario A. The size of this graph was modified in different steps. For each problem size, memory consumption and replanning time were measured. The number of tree nodes in the Task Roadmap are used to represent memory consumption. All replanning times were measured at random locations before completing the first very task, which is the worst case. Scenario A was scaled in two ways, 1) by changing the number of tasks in each layer of the kit boxes (Figure 8ab), by changing the number of layers in the kit boxes (Figure 8B). Both scaling scenarios indicate an exponential growth of the memory consumption, but at different rates where scaling the number of layers is more advantageous. Scaling the number of layers introduces a lot of precedence constraints, while the scaling of layer sizes introduces very few. The B&B-TRM replanning times are within a few seconds for the included problem sizes. The scalability experiments confirms that memory consumption may be a limitation for the algorithm in some scenarios. Especially for less constrained, scaled up problem scenarios.

## 8 Conclusion and Future Work

We have proposed the concept of Task Roadmaps (TRM) and shown that it is a promising strategy to speed up online replanning of robot tasks, thereby contributing to improved productivity in a dynamic environment. We have presented a strategy to implement Task Roadmaps, using a Robot Task Scheduling Graph to model a robot application, Branch and Bound (B&B) for initial planning and B&B-TRM for replanning. The benefits, as well as the limitations for this strategy, have been investigated in an experimental study where a MILP solver and a PDDL based planner have been used as benchmarks.

Future work will address the combining of different replanning strategies with the modelling and runtime observation of disturbance behaviours. Another interesting extension is to widen the scope to multi-robot task allocation and scheduling.

## Data Availability

The original contributions presented in the study are included in the article, further inquiries can be directed to the corresponding author.
