# Corrigendum: Task Roadmaps: Speeding Up Task Replanning

**DOI:** 10.3389/frobt.2022.940811

**Published:** 2022-06-30

**Authors:** Anders Lager, Giacomo Spampinato, Alessandro V. Papadopoulos, Thomas Nolte

**Affiliations:** ^1^ Mälardalen University, Västerås, Sweden; ^2^ ABB AB, Västerås, Sweden

**Keywords:** autonomous robots, task planning, optimization, ROS, robot task modelling

In the original article, Listings 1 and 2 were not included during the typesetting process and were overlooked during production. The missing listings appear below.

Listing 1 PDDL domain

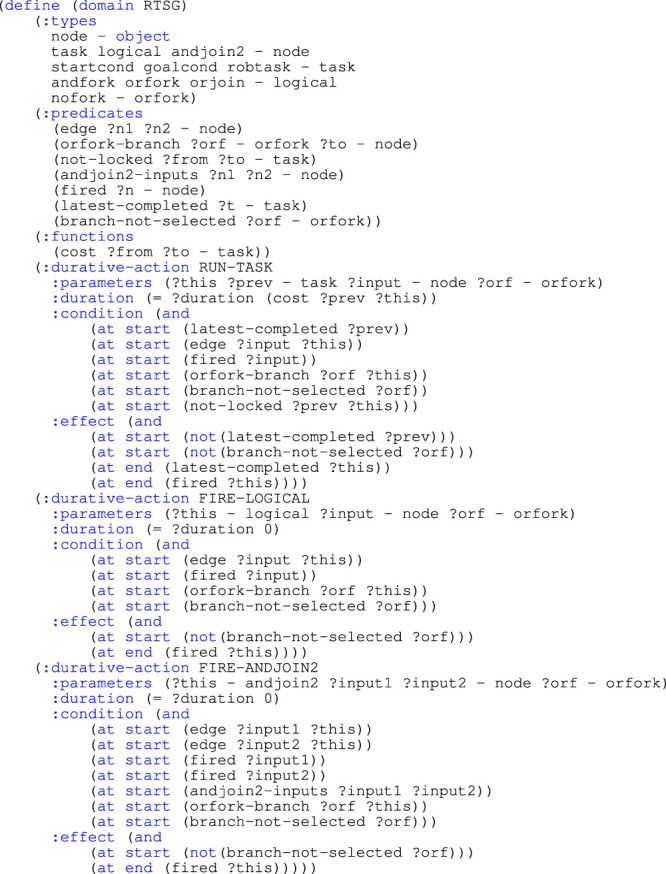

Listing 2. PDDL problem

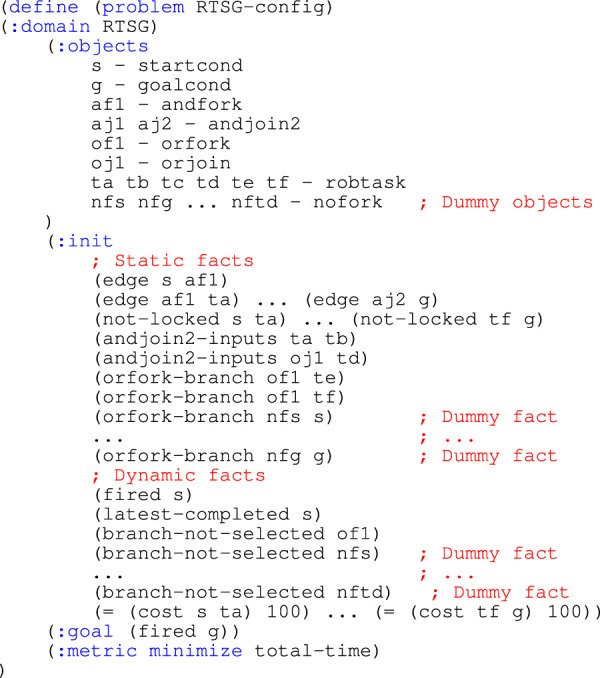

The authors apologize for this error and state that this does not change the scientific conclusions of the article in any way. The original article has been updated.

